# Evaluation of FAPI PET imaging in gastric cancer: a systematic review and meta-analysis

**DOI:** 10.7150/thno.88335

**Published:** 2023-08-21

**Authors:** Dan Ruan, Liang Zhao, Jiayu Cai, Weizhi Xu, Long Sun, Jiayi Li, Jingjing Zhang, Xiaoyuan Chen, Haojun Chen

**Affiliations:** 1 Department of Nuclear Medicine and Minnan PET Center, Xiamen Key Laboratory of Radiopharmaceuticals, the First Affiliated Hospital of Xiamen University, School of Medicine, Xiamen University, Xiamen, China.; 2 The School of Clinical Medicine, Fujian Medical University, Fuzhou, 350004, China.; 3 Department of Medical Oncology, the First Affiliated Hospital of Xiamen University, Xiamen, China.; 4 Departments of Diagnostic Radiology, Yong Loo Lin School of Medicine and Faculty of Engineering, National University of Singapore, Singapore.; 5 Clinical Imaging Research Centre, Centre for Translational Medicine, Yong Loo Lin School of Medicine, National University of Singapore.; 6 Nanomedicine Translational Research Program, NUS Center for Nanomedicine, Yong Loo Lin School of Medicine, National University of Singapore.; 7 Xiamen Key Laboratory of Rare Earth Photoelectric Functional Materials, Xiamen Institute of Rare Earth Materials, Haixi Institute, Chinese Academy of Sciences, Xiamen, China.

**Keywords:** fibroblast activation protein, ^68^Ga-FAPI, ^18^F-FDG, gastric cancer, PET/CT

## Abstract

**Purpose:** Recent studies suggest that ^68^Ga-FAPI PET/CT demonstrated superiority over ^18^F-FDG PET/CT in the evaluation of various cancer types, especially in gastric cancer (GC). By comprehensively reviewing and analysing the differences between ^68^Ga-FAPI and ^18^F-FDG in GC, some evidence is provided to foster the broader clinical application of FAPI PET imaging.

**Methods:** In this review, studies published up to July 3, 2023, that employed radionuclide labelled FAPI as a diagnostic radiotracer for PET in GC were analysed. These studies were sourced from both the PubMed and Web of Science databases. Our statistical analysis involved a bivariate meta-analysis of the diagnostic data and a meta-analysis of the quantitative metrics. These were performed using R language.

**Results:** The meta-analysis included 14 studies, with 527 patients, of which 358 were diagnosed with GC. Overall, ^68^Ga-FAPI showed higher pooled sensitivity (0.84 [95% CI 0.67-0.94] *vs.* 0.46 [95% CI 0.32-0.60]), specificity (0.91 [95% CI 0.76-0.98] *vs.* 0.88 [95% CI 0.74-0.96]) and area under the curve (AUC) (0.92 [95% CI 0.77-0.98] *vs.* 0.52 [95% CI 0.38-0.86]) than ^18^F-FDG. The evidence showed superior pooled sensitivities of ^68^Ga-FAPI PET over ^18^F-FDG for primary tumours, local recurrence, lymph node metastases, distant metastases, and peritoneal metastases. Furthermore, ^68^Ga-FAPI PET provided higher maximum standardized uptake value (SUVmax) and tumour-to-background ratios (TBR). For bone metastases, while ^68^Ga-FAPI PET demonstrated slightly lower patient-based pooled sensitivity (0.93 *vs.* 1.00), it significantly outperformed ^18^F-FDG in the lesion-based analysis (0.95 *vs.* 0.65). However, SUVmax (mean difference [MD] 1.79 [95% CI -3.87-7.45]) and TBR (MD 5.01 [95% CI -0.78-10.80]) of bone metastases showed no significant difference between ^68^Ga-FAPI PET/CT and ^18^F-FDG PET/CT.

**Conclusion:** Compared with ^18^F-FDG, ^68^Ga-FAPI PET imaging showed improved diagnostic accuracy in the evaluation of GC. It can be effectively applied to the early diagnosis, initial staging, and detection of recurrence/metastases of GC. ^68^Ga-FAPI may have the potential of replacing ^18^F-FDG in GC in future applications.

## Introduction

Gastric cancer (GC), a highly prevalent malignancy globally, ranks fifth in incidence and fourth in mortality 1. Adenocarcinoma, constituting over 95% of GC cases, predominates the pathological subtype 2. According to Lauren's classification, which is characterized by the histological structure, GC can be divided into intestinal type and diffuse type 3. Intestinal type cancer originates from the intestinal metaplasia mucosa, while diffuse type cancer originates from the intrinsic mucosa of the stomach. In general, the prognosis for GC of the intestinal type is considered better than that for the diffuse type 4, 5. A discouraging 5-year survival rate of less than 40% underscores an urgent need for more efficacious diagnostic tools and treatment strategies 6, 7. Current multidisciplinary treatments for GC include surgery and systemic therapy (*e.g.* chemotherapy, radiotherapy, targeted therapy and immunotherapy) 8. However, the efficacy of these strategies relies heavily on precise disease assessment, reinforcing the indispensability of proficient diagnostic tools.

One such commonly used diagnostic tool, ^18^F-flouro-deoxy-glucose positron emission tomography/computed tomography (^18^F-FDG PET/CT), finds extensive application in diagnosing, staging, and preoperative evaluation of numerous cancers. For primary GC, the diagnostic sensitivity of ^18^F-FDG PET has varied substantially, from 26% for early-stage to 95% for advanced GC. Tumours <30 mm displayed a sensitivity as low as 17% 9, 10. Moreover, certain pathologies, such as non-intestinal-type gastric adenocarcinoma (signet ring cell carcinoma (SRCC) and mucinous adenocarcinoma), show limited ^18^F-FDG uptake leading to low ^18^F-FDG PET/CT sensitivity 11. This results in significant reduction in diagnostic specificity due to masking of the primary or recurrent tumour by physiological ^18^F-FDG uptake in the normal gastric wall 12, 13. Additionally, gastritis may produce false-positive results on ^18^F-FDG PET/CT 14, 15. The diagnostic and prognostic implications of GC patients are critically reliant on accurate staging, yet, ^18^F-FDG PET often falls short in sensitivity when evaluating lymph node infiltration and distant metastasis 16, 17.

Within the tumour microenvironment, cancer-associated fibroblasts (CAFs), an integral part of stroma, contribute significantly to cancer initiation, progression, and metastasis 18-20. Fibroblast activator protein (FAP), a member of the S9B subfamily of serine protease, is primarily expressed by CAFs. FAP is detected in primary and metastatic cancers of various organs (including colorectal, breast, ovarian, bladder, lung, *etc.*), yet is virtually absent in normal adult tissues 21. Capitalizing on this distinctive attribute of tumour stroma, radiotracers comprised of FAP inhibitors (FAPIs) have been developed for imaging of various malignancies 22. Since 2018, researchers have employed DOTA-chelated FAPI with gallium 68 (^68^Ga) for diagnosing various tumours using PET 23. Unlike ^18^F-FDG, which reflects the glucose metabolism of tumour cells, radiolabelled FAPI PET imaging exposes CAFs and extracellular fibrosis in the tumour stroma. Notably, FAP is highly expressed in GC's CAFs but absent in quiescent fibroblasts or healthy adult tissues 24. Moreover, the expression levels of FAP in CAFs are significantly correlated with Lauren's classification, degree of differentiation, depth of tumour invasion, and TNM stage 24. Currently, FAPI PET/CT presents a more distinct tumour profile and higher tumour-to-background ratio (TBR) than ^18^F-FDG for GC imaging 25. However, it should be noted that radiolabelled FAPI molecule is not yet approved by the US Food and Drug Administration (FDA) or the European Medicines Agency (EMA) and is still in Phase III clinical trials.

Given the limitations of ^18^F-FDG PET in GC diagnosis, this review aims to comprehensively evaluate the merits of FAPI PET in early diagnosis, initial staging, and detection of recurrence/metastases in GC. This includes original studies on ^68^Ga-FAPI PET/CT or positron emission tomography/magnetic resonance (PET/MR), thus providing substantial evidence to promote the future clinical application of radionuclide labelled FAPI.

## Methods

### Search strategy and study selection

In accordance with the Preferred Reporting Items for Systemic Reviews and Meta-Analysis (PRISMA) 2020 statement flow, we selected publications that fulfilled specific criteria (registration number, PROSPERO CRD42023447654). We conducted a comprehensive search of the primary databases, PubMed and Web of Science, for publications from 1 January 2018 to 3 July 2023. The search strategy incorporated keywords such as (FAPI OR 'fibroblast activation protein') AND ('gastric' OR 'stomach'). Our review targeted studies exploring the diagnostic application of radionuclide labelled FAPI in GC, focusing on diagnosing primary tumours, recurrent tumours, metastatic lymph nodes, bone/visceral metastases, and peritoneal metastases. Studies without quantitative assessment parameters (SUVmax or TBR) or diagnostic data were excluded, as were reviews, case reports, commentaries, and editorials. We applied conditional filters progressively in line with the PubMed and Web of Science search rules to streamline our search results. We meticulously reviewed the titles, abstracts, full texts, and supplementary materials of publications for relevance. Two independent reviewers participated in the study selection process to mitigate bias and in instances where disagreements arose during the study inclusion and data extraction process, these differences were reconciled through thorough discussion, reaching consensus by consulting a third reviewer if necessary.

### Data collection process

We compiled relevant data from the selected studies, including fundamental details such as authors, year of publication, patient source, study design, primary aim, and the patient count. Furthermore, we extracted data from the 'Methods' and 'Results' sections, comprising details on the radiotracer type, imaging modality, reference standard, interval between FDG PET and FAPI PET, method of image analysis, duration from radiotracer injection to examination, and quantitative assessment metrics. The extracted data covered GC type, patient composition, patient age, sex, diagnosis-related data (True positive (TP) / False positive (FP) / false negative (FN) / True negative (TN)), and quantitative assessment parameters (SUVmax or TBR). Data extraction was carried out independently by two reviewers.

### Risk of bias and quality assessment

We utilized the Quality Assessment of Diagnostic Accuracy Studies (QUADAS-2) tool in Review Manager (RevMan) software (RevMan for Windows, version 5.4.1, Developed by the Cochrane Collaboration) to assess the risk of bias and applicability for each study, from which the software derived comprehensive assessment metrics. The main items we assessed included patient selection, index test, reference standard, and flow and timing.

### Data analysis

Bivariate meta-analysis of diagnostic data (TP / FP/ FN / TN) was performed using the 'meta4diag' package (RRID:SCR_023024) of the R project (R for Windows, version 4.1.0) to obtain pooled sensitivities and specificities and fitted summary receiver-operation characteristic (SROC) curves. This method is a Bayesian bivariate analysis based on the integrated nested Laplace approximation method, which fully takes into account the heterogeneity among studies and the correlations between sensitivity and specificity. We calculated study sensitivities and generated forest plots using RevMan (version 5.4.1, Developed by the Cochrane Collaboration). Moreover, pooled sensitivities and 95% confidence intervals (CI) were calculated for each subgroup using Meta-Disc software (version 1.4). The R project was used to analyse quantitative analysis parameters (SUVmax or TBR), with the 'meta' (RRID:SCR_019055) and 'metafor' (RRID:SCR_003450) packages loaded to calculate pooled Mean difference (MD) and 95% CI. A random effects model was adopted if I^2^ >50% or p <0.05, whereas a fixed-effects model was adopted if I^2^ <50% or p >0.05 26. We created funnel plots based on SUVmax- and TBR-based analysis results to evaluate publication bias and heterogeneity. Publication bias was further analysed using Begg's test. Meta-analyses for both SUVmax and TBR utilized means and standard deviations (SD); hence, the median and range reported in the studies were converted using the methods of Luo *et al.* (2018) 27 and Wan *et al.* (2014) 28. For studies in which multiple mean and standard deviation sets needed to be combined as a set of values, we used Equations (1) 
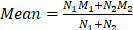
 , and (2) 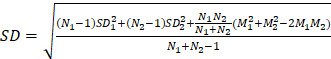 ; (N_1_ and N_2_ are the sample sizes, M_1_ and M_2_ are the means, and SD_1_ and SD_2_ are the standard deviations).

## Results

### Study selection

**Figure 1:**
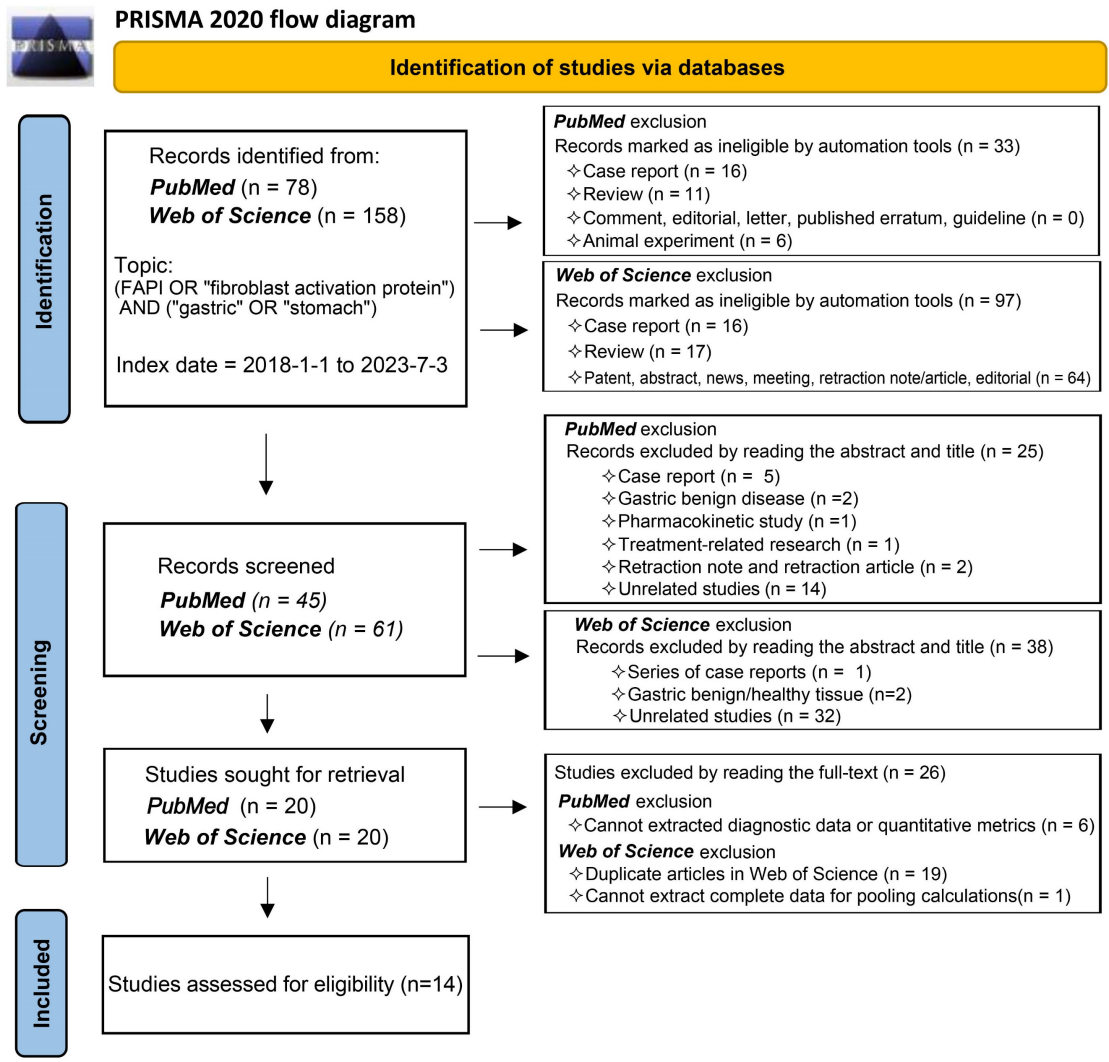
Flowchart of the study inclusion process.

Our comprehensive search process led to the inclusion of 14 studies 25, 29-41. One study investigating the role of ^18^F-labelled FAPIs in primary tumours and peritoneal metastasis of GC was excluded due to its retraction 42. As a result, all remaining studies utilized ^68^Ga-labelled FAPI as the radiotracer. The search process is comprehensively outlined in **Figure 1**.

### Study characteristics

A total of 527 patients were included in the studies, with 358 diagnosed with GC. Patients' mean or median ages ranged from 51-70 years, with a gender ratio of 315:212 favouring males. Eight out of the 14 studies were prospective in design, with most of the patients originating from China. The studies aimed to compare the role of ^68^Ga-FAPI with ^18^F-FDG PET in the initial staging and detection of recurrence/metastases in GC. Ten out of the 14 studies combined pathology with clinical follow up as the reference standard. Most studies were designed with a 1-week interval between the two imaging modalities, and most PET scans were typically conducted approximately 60 minutes post-radiotracer injection. Nine of the 14 studies quantified GC-related lesion parameters. Notably, two studies used PET/CT with PET/MR for imaging, and two studies used PET/MR exclusively. Except for the study by Chen *et al.* which included only SRCC 39, GC's pathological types in the other studies included adenocarcinomas with varying degrees of differentiation and SRCCs (**Table 1**). The comprehensive data utilized for the diagnostic accuracy analysis of GC is presented in **Table S1**.

### Methodological qualitative assessment

**Figure 2:**
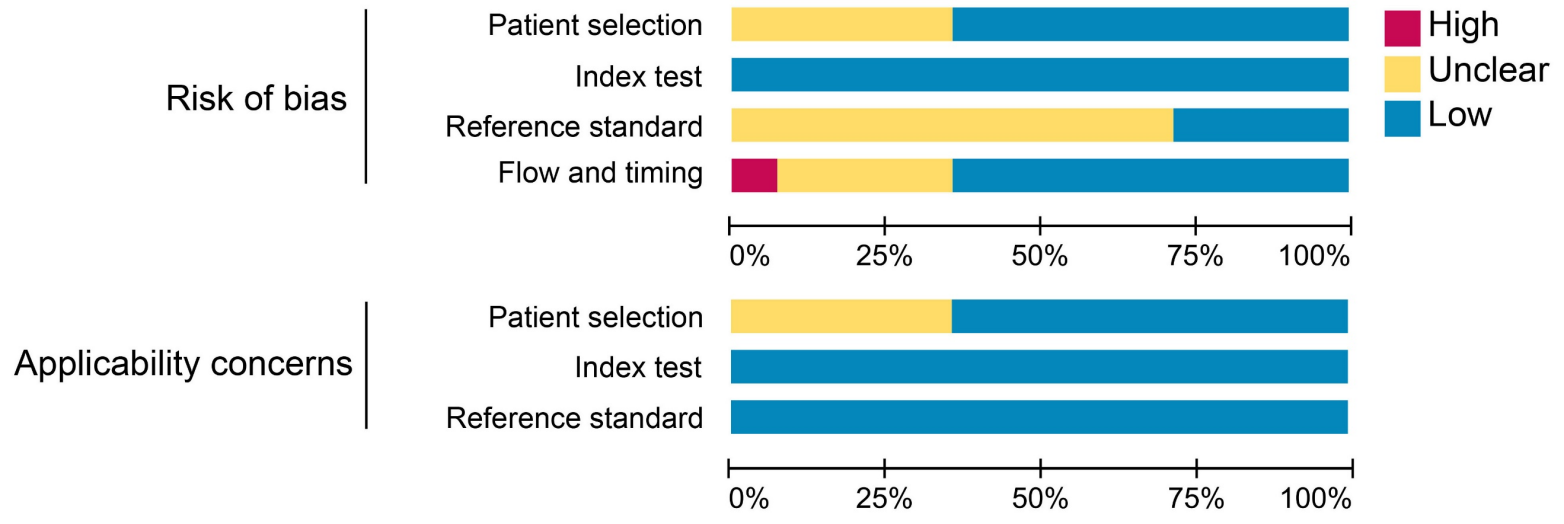
Summary assessment of risk of bias and applicability concerns of included studies.

The results of the quality assessment of each study are detailed in **Figure S1**. One study was unanimously adjudicated as high risk due to 2 tracers applied at intervals of 2 weeks or more 29. Some studies had uncertain risk due to the inclusion of other malignancies or inconsistent reference standards. Overall, about 36% of the studies had uncertain risk bias in patient selection; no studies had risk bias in the index test; roughly 71% of the studies had uncertain bias in reference standards; and 7% and 29% of the studies had a high and uncertain risk of bias in flow and timing, respectively. Regarding clinical applicability, no studies were deemed highly inapplicable, but 36% had uncertainty in patient selection (**Figure 2**).

### Overall pooled diagnostic accuracy of ^68^Ga-FAPI and ^18^F-FDG

**Figure 3:**
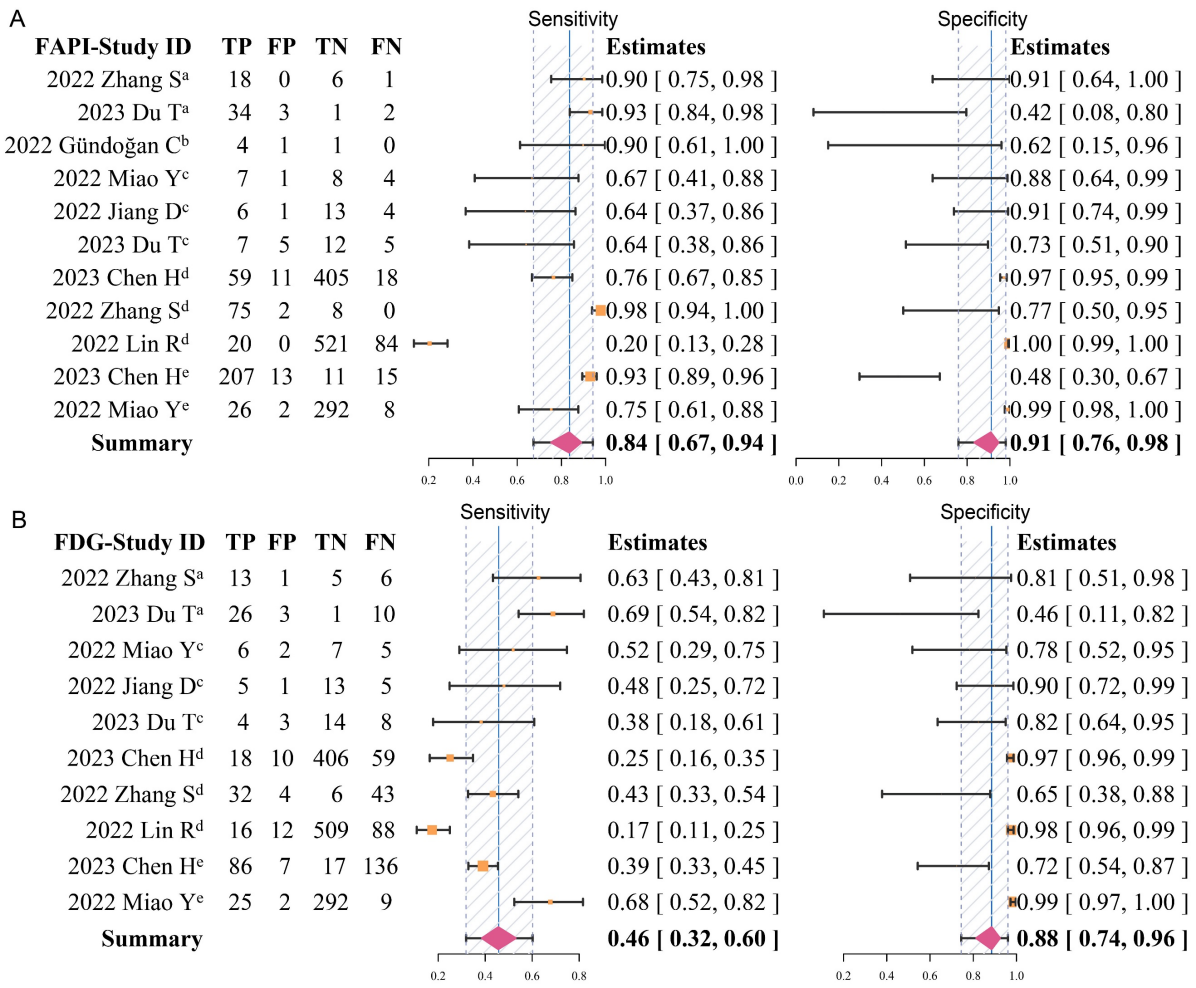
Forest plots of diagnostic sensitivity and specificity of ^68^Ga-FAPI (A) and ^18^F-FDG (B) (Data sets extracted from patient-based studies: ^a^Primary tumour, ^b^recurrent tumour, ^c^metastatic lymph nodes; Data sets extracted from lesion-based studies: ^d^metastatic lymph nodes, ^e^distant metastases).

**Figure 4:**
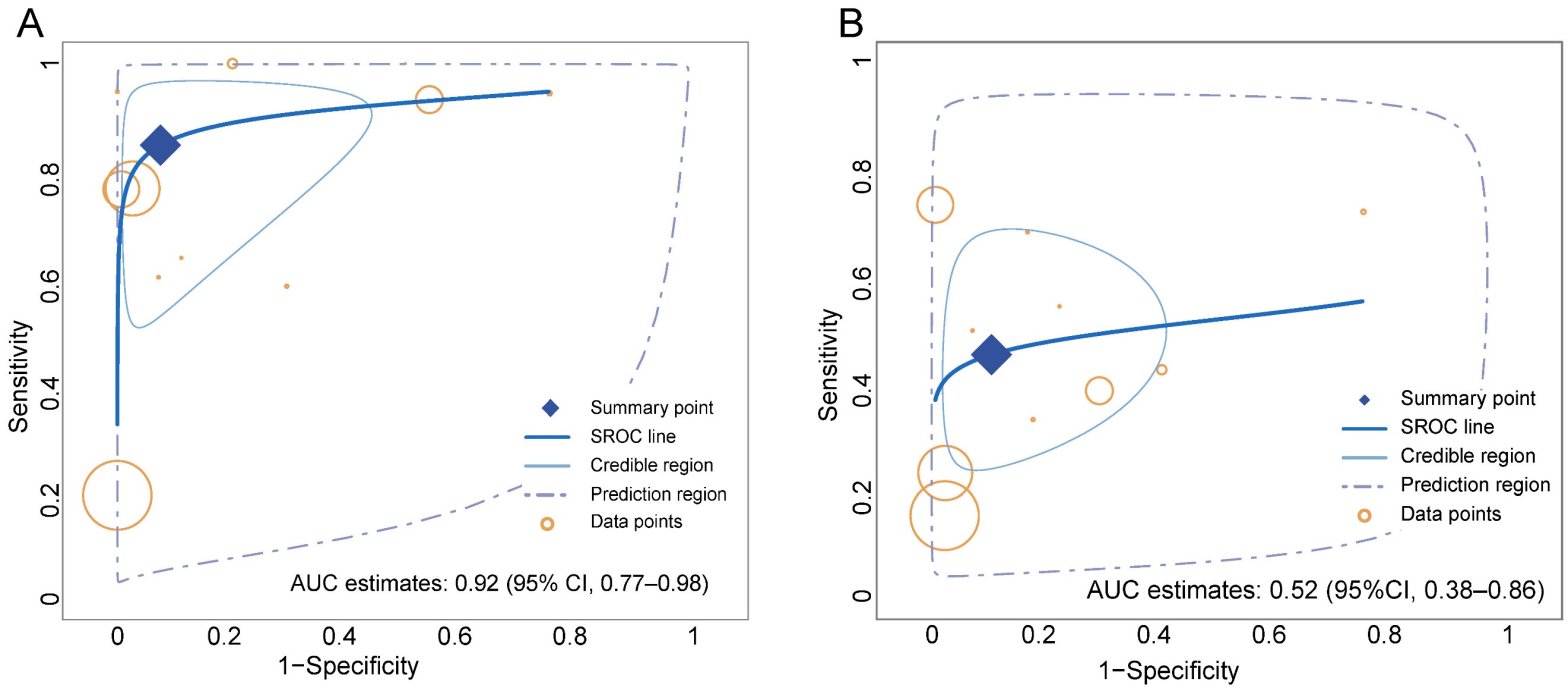
Summary receiver-operation characteristic (SROC) curves for the overall performance assessment of ^68^Ga-FAPI (A) and ^18^F-FDG (B) for GC (gastric cancer).

Overall, the pooled sensitivity (0.84 [95% CI 0.67-0.94] *vs.* 0.46 [95% CI 0.32-0.60]) of ^68^Ga-FAPI in GC was significantly higher than that of ^18^F-FDG, and the pooled specificity (0.91 [95% CI 0.76-0.98] *vs.* 0.88 [95% CI 0.74-0.96]) was slightly higher than that of ^18^F-FDG (**Figure 3**). In addition, the SROC curves fitted on the basis of the sensitivity and specificity of ^68^Ga-FAPI showed that the curve and its summary point were located at the upper left corner of the coordinate axis, and the area under the curve (AUC) reached 0.92 (95% CI 0.77-0.98), indicating the excellent diagnostic efficacy of ^68^Ga-FAPI. However, the ^18^F-FDG-based SROC curves showed a more discrete distribution of data points, and the location of the curve and its summary point were far from the upper left corner. Therefore, the overall shape of the SROC curve and the AUC (0.52 [95% CI 0.38-0.86]) indicated the poor diagnostic performance of ^18^F-FDG in GC (**Figure 4**).

### Pooled sensitivity analysis of subgroups

In patient-based analysis, ^68^Ga-FAPI PET displayed superior sensitivity to ^18^F-FDG in the diagnosing primary tumours (0.95 [95% CI 0.91-0.97] *vs.* 0.72 [95% CI 0.67-0.78]), recurrent tumours (1.00 [95% CI 0.89-1.00] *vs.* 0.48 [95% CI 0.29-0.68]), lymph node metastases (0.72 [95% CI 0.59-0.82] *vs.* 0.47 [95% CI 0.32-0.62]), distant metastases (0.83 [95% CI 0.59-0.96] *vs.* 0.50 [95% CI 0.26-0.74]), and peritoneal metastases (0.98 [95% CI 0.92-1.00] *vs.* 0.47 [95% CI 0.34-0.59]) (**Table 2**). Four studies reported suboptimal sensitivity of ^68^Ga-FAPI PET in diagnosing of lymph node metastases, with sensitivity values below 65% 33, 35, 36, 40. Miao *et al.* revealed a significantly lower sensitivity for ^68^Ga-FAPI PET than for ^18^F-FDG when diagnosing bone metastases, potentially leading to an overall reduced pooled sensitivity (0.93 [95% CI 0.66-1.00] *vs.* 1.00 [95% CI 0.77-1.00]) for ^68^Ga-FAPI than ^18^F-FDG 36. Although both Gündoğan *et al.* and Qin *et al.* reported the same patient-based sensitivity of ^68^Ga-FAPI and ^18^F-FDG PET/CT in cases of bone metastases, ^68^Ga-FAPI PET/CT detected more bone lesions than ^18^F-FDG 32, 37. The comparative sensitivities of ^68^Ga-FAPI and ^18^F-FDG are graphically represented in **Figure S2**.

On performing a lesion-based analysis, each study showed superior sensitivity of ^68^Ga-FAPI over ^18^F-FDG (**Figure S3**). As anticipated, the pooled sensitivities of ^68^Ga-FAPI PET in diagnosing metastatic lymph nodes (0.64 [95% CI 0.58-0.70] *vs.* 0.32 [95% CI 0.27-0.38]), visceral metastases (0.96 [95% CI 0.94-0.97] *vs.* 0.45 [95% CI 0.41-0.48]), bone metastases (0.95 [95% CI 0.91-0.98] *vs.* 0.65 [95% CI 0.57-0.71]), and peritoneal metastases (1.00 [95% CI 0.98-1.00] *vs.* 0.31 [95% CI 0.25-0.37]) were significantly better than that of ^18^F-FDG (**Table 2**). Interestingly, Lin *et al.* reported an extremely low sensitivity for both ^68^Ga-FAPI and ^18^F-FDG PET/CT in the diagnosis of lymph nodes (0.19 *vs.* 0.15) 35. In the diagnosing distant metastases, both radiotracers demonstrated slightly suboptimal sensitivity but high specificity as reported by Miao *et al.* 36.

### Diagnostic accuracy with dual-tracer PET/CT

In detecting GC recurrence, Gündoğan *et al.* identified a false-positive ^68^Ga-FAPI uptake in a patient (1/2) with no recurrence but was correctly diagnosed by ^18^F-FDG PET 32. Therefore, dual-tracer PET/CT could enhance the specificity of diagnosing recurrent tumours compared to ^68^Ga-FAPI alone (specificity: 0.50 to 1.00). In Qin *et al.*'s study, ^18^F-FDG aided in reducing false-negative lymph nodes diagnosed on ^68^Ga-FAPI PET, suggesting that combining ^18^F-FDG and ^68^Ga-FAPI PET could be more effective in differentiating lymph node infiltration from non-infiltration (sensitivity shifted from 0.88 to 0.94) 37. Miao* et al.*'s study demonstrated that the use of dual-tracer PET/CT (^68^Ga-FAPI + ^18^F-FDG) significantly increased the sensitivities in diagnosing lymph node metastases (0.64 to 0.73), distant metastases (0.76 to 0.97), bone metastases (0.67 to 1.00), liver metastases (0.57 to 1.00), and lung metastases (0.00 to 1.00) 36. Overall, ^18^F-FDG PET may provide valuable insights to ^68^Ga-FAPI PET in diagnosing lymph node and distant metastases, particularly for bone, liver, and lung metastases (**Table 3**).

### Quantitative parameter analysis of ^68^Ga-FAPI and ^18^F-FDG

**Figure 5:**
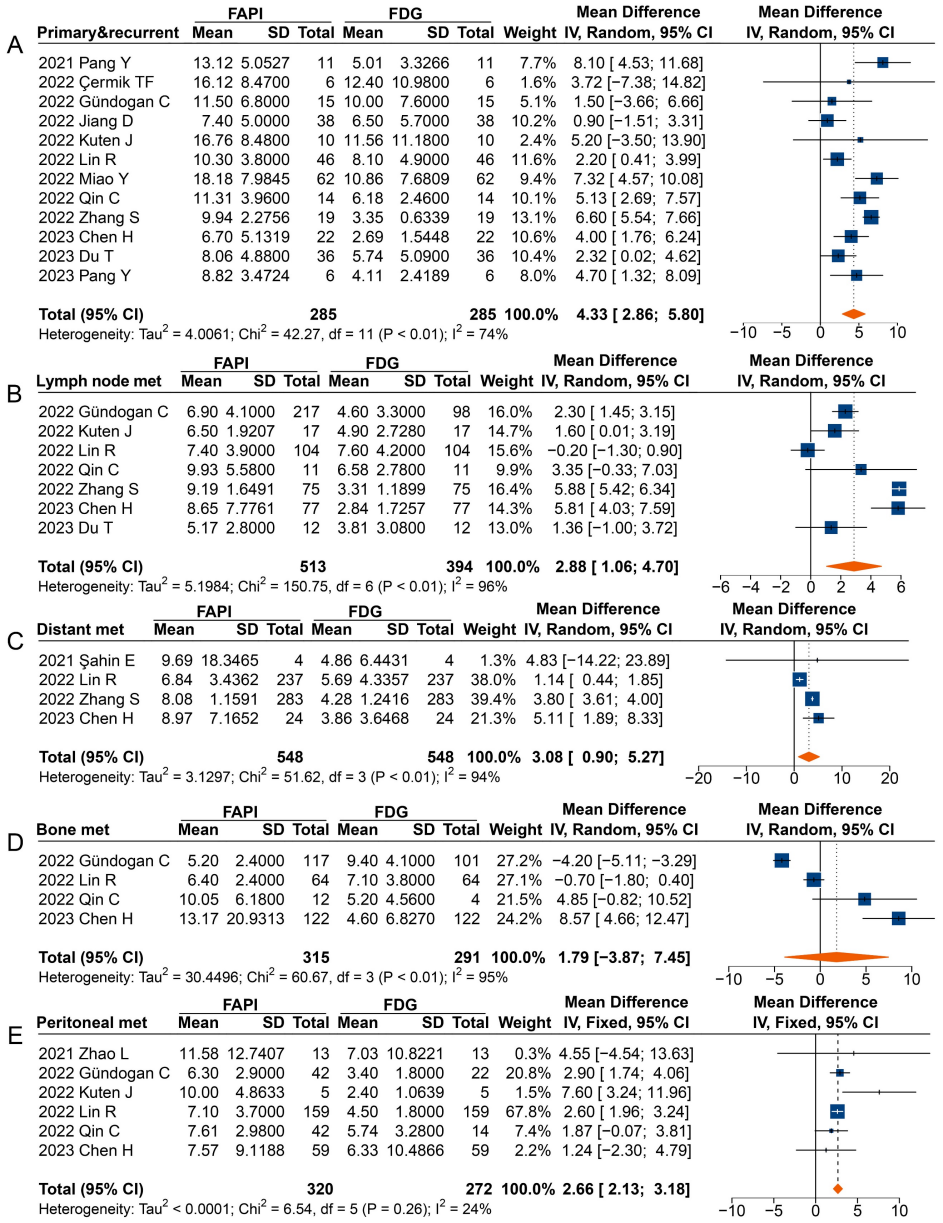
Forest plots comparing the uptake values of ^68^Ga-FAPI and ^18^F-FDG (SUVmax) for primary and recurrent tumours (A), metastatic lymph nodes (B), distant metastases (C), bone metastases (D), and peritoneal metastases (E). There was significant heterogeneity when the SUVmax data sets for primary and recurrent tumours (I^2^ = 74%), metastatic lymph nodes (I^2^ = 96%), distant metastases (I^2^ = 94%), and bone metastases (I^2^ = 95%) were pooled, so a random-effects model was used. Heterogeneity was not apparent when SUVmax data sets were pooled for peritoneal metastases (I^2^ = 24%), so a fixed-effects model was used.

In analysing primary and recurrent tumours (MD 4.33 [95% CI 2.86-5.80]), metastatic lymph nodes (MD 2.88 [95% CI 1.06-4.70]), distant metastases (MD 3.08 [95% CI 0.90-5.27]), and peritoneal metastases (MD 2.66 [95% CI 2.13-3.18]), a significantly higher uptake of ^68^Ga-FAPI was observed comparted to ^18^F-FDG (**Figure 5**). However, in the analysis of primary or recurrent tumours, four studies found no difference between ^68^Ga-FAPI and ^18^F-FDG uptake 31-34.

Most studies reported a higher SUVmax derived from ^68^Ga-FAPI PET than that from ^18^F-FDG in analysing radiotracer uptake in metastatic lymph nodes, with three studies not reporting significant differences 35, 37, 40. When analysing distant metastases, only Şahin *et al.* found no difference in radiotracer uptake between ^68^Ga-FAPI and ^18^F-FDG 29. Specifically, the overall analysis of peritoneal metastases (MD 2.66 [95% CI 2.13-3.18]) showed significantly higher uptake of ^68^Ga-FAPI than ^18^F-FDG by the lesions, despite three studies finding no difference between the SUV measured on ^68^Ga-FAPI PET and ^18^F-FDG PET 30, 37, 39. However, no significant differences were found in the SUVmax between ^68^Ga-FAPI and ^18^F-FDG PET imaging for bone metastases (MD 1.79 [95% CI -3.87-7.45]).

**Figure 6:**
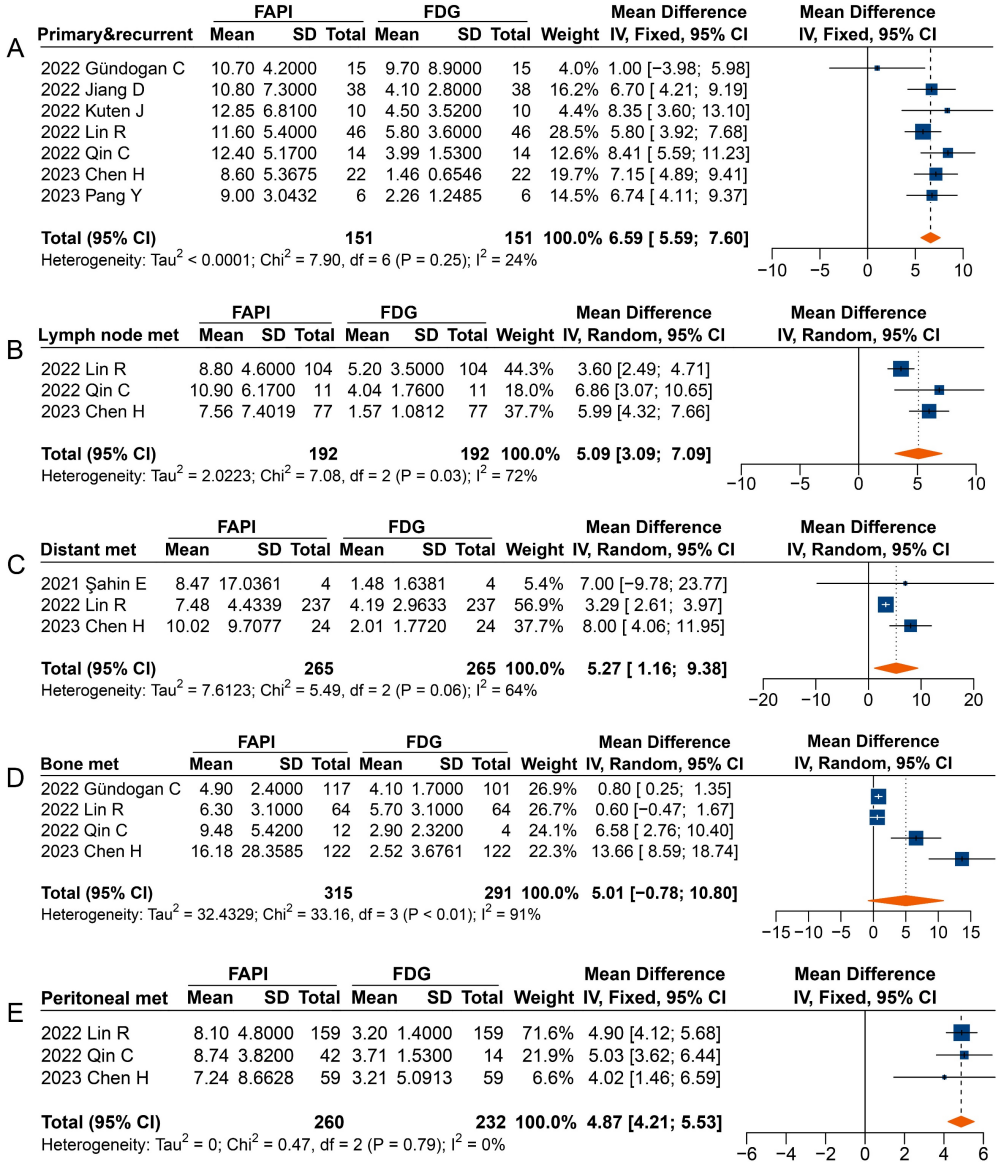
Forest plot comparing the TBR of primary and recurrent tumours (A), metastatic lymph nodes (B), distant metastases (C), bone metastases (D), and peritoneal metastases (E) between ^68^Ga-FAPI and ^18^F-FDG PET imaging. There was significant heterogeneity when the TBR data sets for metastatic lymph nodes (I^2^ = 72%), distant metastases (I^2^ = 64%), and bone metastases (I^2^ = 91%) were pooled, so a random-effects model was used. There was no significant heterogeneity when the SUVmax data sets for primary and recurrent tumours (I^2^ = 24%) and peritoneal metastases (I^2^ = 0%) were pooled, so a fixed-effects model was used.

Overall, significantly higher TBRs were measured with ^68^Ga-FAPI PET than those with ^18^F-FDG PET for primary and recurrent tumours (MD 6.59 [95% CI 5.59-7.60]), metastatic lymph nodes (MD 5.09 [95% CI 3.09-7.09]), distant metastases (MD 5.27 [95% CI 1.16-9.38]), and peritoneal metastases (MD 4.87 [95% CI 4.21-5.53]) (**Figure 6**). No significant difference was observed in the TBR of bone metastases (MD 5.01 [95% CI -0.78-10.80]) measured between ^68^Ga-FAPI and ^18^F-FDG PET. In terms of primary tumours or recurrence detection, only one study showed no difference in TBR between ^68^Ga-FAPI and ^18^F-FDG PET 32. Regarding distant metastases, Şahin *et al.* observed no difference in TBR measured by ^68^Ga-FAPI and ^18^F-FDG PET 29.

### Publication bias

**Figure 7:**
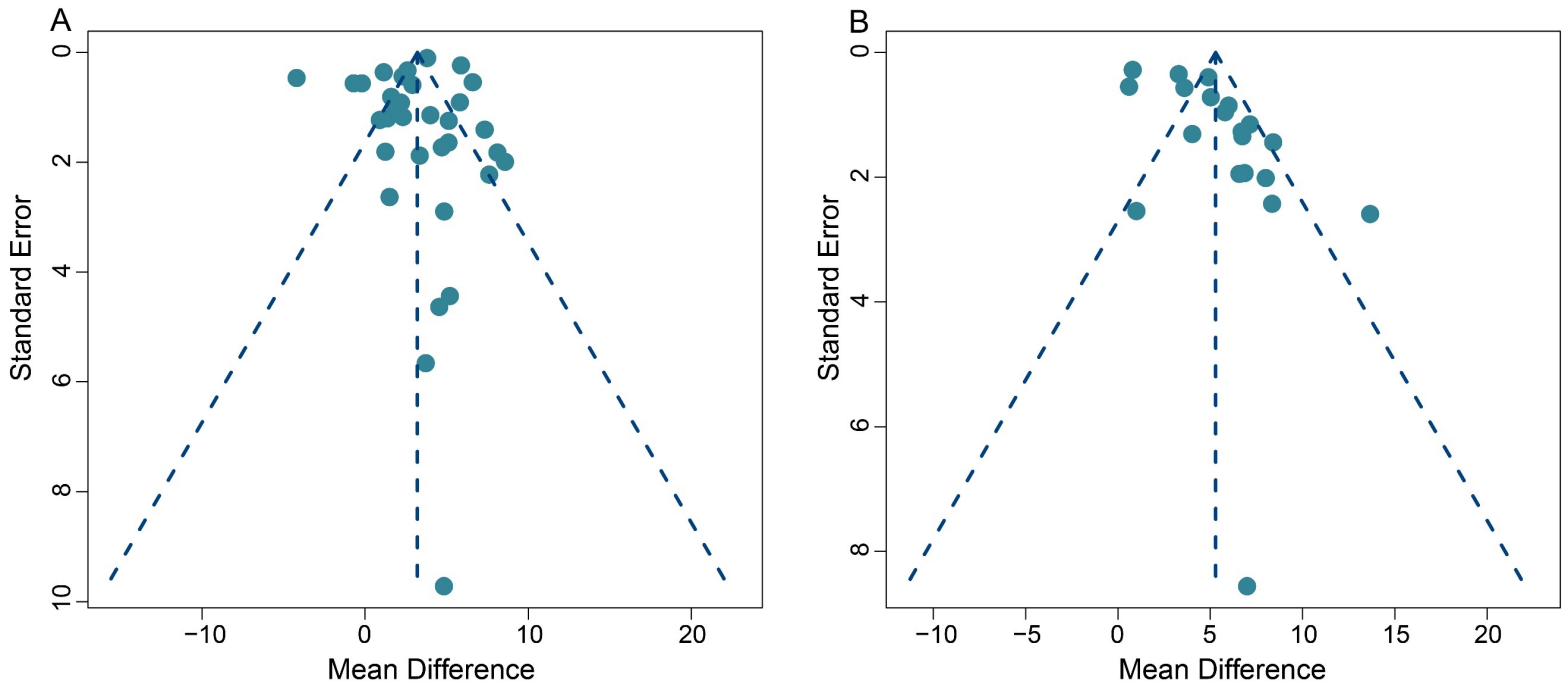
Funnel plots for SUVmax-based analysis (A) and TBR-based analysis (B).

This meta-analysis exhibited some heterogeneity within the pooled SUVmax data. This is demonstrated by the fact that 10 out of 33 (30.3%) data sets fell outside the 95% CI in the funnel plot (**Figure 7A**). Additionally, five out of 20 (25.0%) data sets also appeared outside the 95% CI in the funnel plot, when we conducted the TBR analysis, indicating some heterogeneity (**Figure 7B**).

On a positive note, the majority of our pooled studies are situated at the apex of the funnel plot. This position indicates a large data sample size, which bolsters the reliability and stability of our pooled results. A comprehensive view of the funnel plot reveals that the data from all studies are distributed in a roughly even manner on both sides of the effect values. This distribution suggests the absence of significant publication bias in both our SUVmax-based and TBR-based meta-analyses.

Furthermore, Begg's test results (z = 1.49, p = 0.14 for SUVmax; z = 0.39, p = 0.70 for TBR) provided no evidence of significant publication bias in this meta-analysis, and further supports the robustness of our collective results.

## Discussion

An optimal imaging modality is crucial for early diagnosis and accurate staging in patients with GC. Contrast-enhanced CT (CE-CT) and MRI are widely recommended for diagnosing GC. However, the diagnosis of GC by CE-CT is influenced by morphological features, histological type, gastric wall thickness, and contrast enhancement patterns. In addition, identification of regional lymph node and distant metastases in CE-CT does not reach satisfactory sensitivity and specificity, particularly for the small liver and peritoneal metastases 43-46. MRI is superior to CE-CT in detecting liver metastases due to its high resolution but is similarly sensitive and specific in detecting lymph node metastases 47-49. Additionally, MRI is limited in detecting distant metastases because of its screening range 50. Although NCCN guideline does not recommend ^18^F-FDG PET/CT as the first-line imaging modality for the diagnosis of GC, it may be considered in high-risk patients to detect distant metastases 2. However, owing to its limited sensitivity for detecting involved lymph nodes, liver, and peritoneal metastases, ^18^F-FDG PET/CT is sometimes of limited use for surgical planning in GC 25, 29, 35, 38, 39.

In recent years, many studies have proposed that ^68^Ga-FAPI could be used as a promising PET tracer for the diagnosis of GC. Our systematic review, which gathers original studies on GC from 2018 onwards, provides strong clinical evidence to support the superior diagnostic sensitivity of ^68^Ga-FAPI over ^18^F-FDG for identifying primary tumours, recurrent tumours, lymph node infiltration, and distant metastases. In contrast to the previous meta-analysis 51, 52, our study brings forth some important nuances and enhancements. For one, we presented the comparative results and a subgroup analysis of the diagnostic accuracy of ^68^Ga-FAPI versus ^18^F-FDG PET/CT in gastric cancer, which was not extensively covered in previous work. Additionally, we calculated and compared the SUVmax and TBRs derived from these two PET scans, providing more depth to our results. Significantly, our study involves a larger patient population than Wang's study 51, encompassing 358 patients as opposed to 148, adding robustness to our findings. We also have observations on the diagnostic accuracy for lymph node metastases. While our patient-based pooled sensitivity mirrored the study by Rizzo *et al.* (0.72 *vs.* 0.74) 52, our lesion-based pooled sensitivity was slightly lower (0.64 *vs.* 0.74), suggesting that further investigation may be warranted. Overall, our results suggest that the diagnostic accuracy of ^68^Ga-FAPI in gastric cancer (GC) was higher than that of ^18^F-FDG. However, in terms of specificity, ^68^Ga-FAPI did not exhibit a significant advantage over ^18^F-FDG. This nuanced understanding of the results positions our work as a valuable contribution to the ongoing research in this field.

Significantly, ^68^Ga-FAPI PET imaging demonstrates a higher sensitivity than ^18^F-FDG for diagnosing primary tumours (0.95 *vs.* 0.72) and locally recurrent tumours (1.00 *vs.* 0.48). This can be largely attributed to the high uptake of ^68^Ga-FAPI in primary or recurrent gastric tumours, coupled with the low background activity of the normal gastric wall. Research has revealed that certain histopathological types of GC, such as SRCC and mucinous carcinoma, have lower ^18^F-FDG avidity than other adenocarcinomas due to relatively low expression levels of glucose transporter 1 (GLUT-1), a fewer number of active cancer cells, and high mucus-containing components 9, 11, 35, 53. In addition, non-intestinal diffuse GCs, which include poorly differentiated adenocarcinoma, SRCC, and non-solid types, infiltrate the gastric wall accompanied by a significant amount of fibrotic tissue but with a low concentration of cancer cells. Given the strong correlation between ^18^F-FDG uptake and the number of active tumour cells, the non-intestinal diffuse type exhibits considerably lower ^18^F-FDG avidity than the intestinal type, which has a higher density of malignant tumour cells 54, 55.

**Figure 8:**
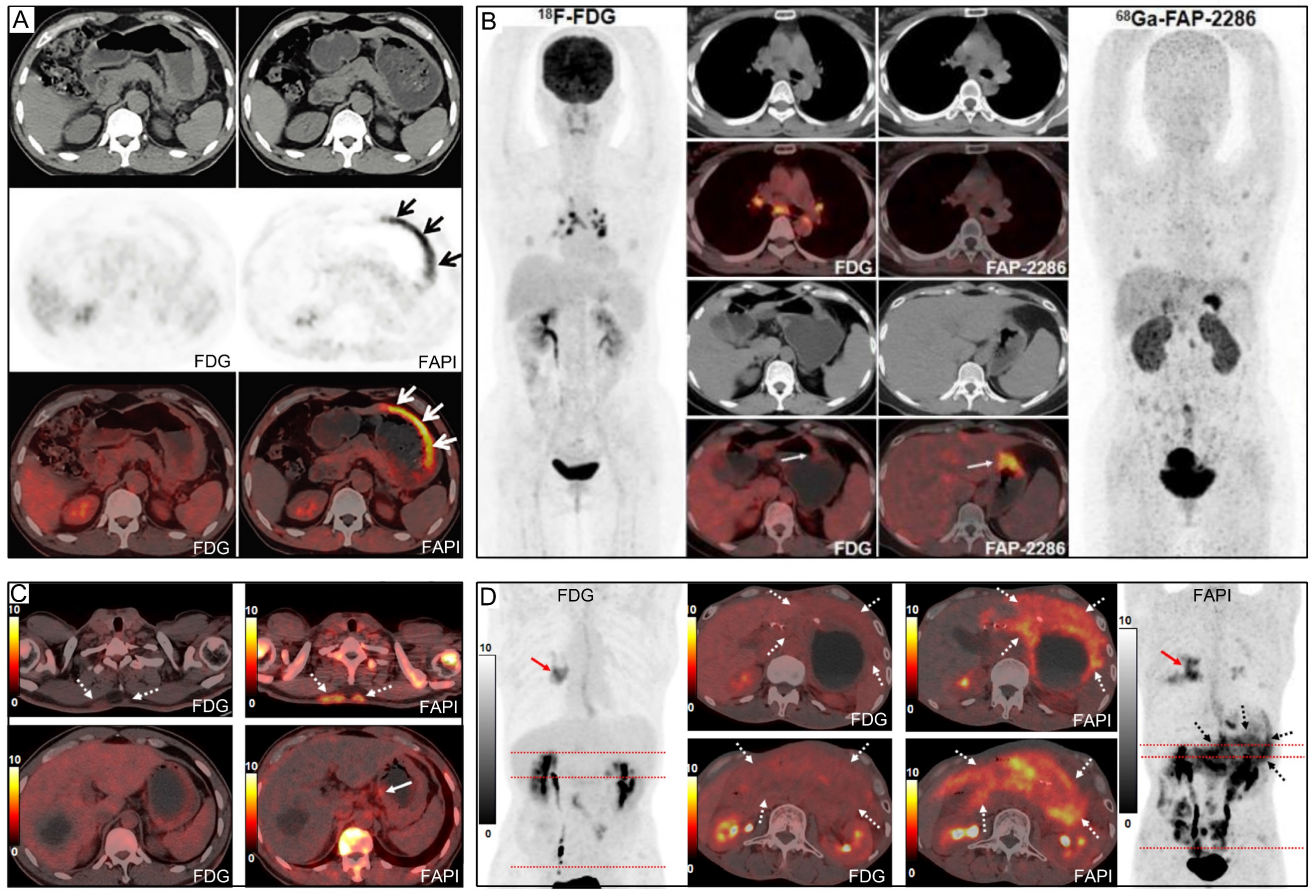
Representative ^18^F-FDG and ^68^Ga-FAPI PET/CT images of primary and metastatic gastric signet ring cell carcinoma (GSRCC). (A) A 55-year-old man with GSRCC underwent ^18^F-FDG-PET/CT for initial staging. ^18^F-FDG PET/CT showed normal findings, while ^68^Ga-FAPI PET/CT revealed intense uptake along the gastric wall. (B) A 40-year-old woman with GSRCC underwent PET/CT for tumour staging. ^68^Ga-FAP-2286 showed higher uptake in the primary tumour than ^18^F-FDG. (C) A 52-year-old man with widespread subcutaneous and bone metastases underwent ^18^F-FDG PET/CT for localizing the primary tumour. However, no intense ^18^F-FDG uptake that likely presenting the primary tumour was observed. ^68^Ga-FAPI PET/CT revealed intense uptake in the lesser curvature of the stomach. A subsequent gastroscopic biopsy confirmed the diagnosis of GSRCC. (D) A 49-year-old man with prior gastrectomy for GSRCC presented with progressive abdominal pain. ^68^Ga-FAPI PET/CT revealed higher radiotracer uptake and larger disease extent than ^18^F-FDG in peritoneal metastases. Source: From Pang, Yizhen *et al.* (2021), Pang, Yizhen *et al.* (2023), and Chen, Haojun *et al.* (2023) with modifications.

On the other hand, advanced SRCC often presents as a 'scirrhous' type of cancer, with a profuse tumour stroma accounting for 90% or more of the cancer mass. The high number of cancer-associated fibroblasts (CAFs) within the tumour stroma, which overexpress fibroblast activation protein (FAP), results in increased ^68^Ga-FAPI uptake at lesion sites 24, 56, 57. Chen's study, which exclusively included patients with SRCC, showed a greater advantage for ^68^Ga-FAPI over ^18^F-FDG in detecting primary and metastatic SRCC (**Figure 8**), although the sensitivity of ^68^Ga-FAPI for SRCC (73%) was significantly lower than that for adenocarcinoma, as reported in other studies 39. Similarly, Gündoğan's study reported that SRCC and mucinous carcinoma demonstrated mild uptake of ^18^F-FDG but intense uptake of ^68^Ga-FAPI 32.

However, it's important to note that ^68^Ga-FAPI has shown limited sensitivity in detecting early-stage GC within the mucosal and submucosal layers, with only 37.5% of primary lesions showing high ^68^Ga-FAPI avidity 36. Chen's study demonstrated that both ^68^Ga-FAPI and ^18^F-FDG missed primary SRCC in 6 of the 22 (27%) patients 39. It was suggested that the number of active tumour cells and stroma accumulating ^18^F-FDG or ^68^Ga-FAPI increased with tumour size. Jiang *et al.* confirmed this result, reporting that the uptake of ^68^Ga-FAPI was lower in small GCs (≤4 cm) than in larger ones (>4 cm) 33. This implies that although ^68^Ga-FAPI has higher sensitivity than ^18^F-FDG in advanced GC, its efficacy in detecting small and early-stage GC needs further investigation in large-scale studies. Furthermore, inflammation, radiotherapy, and surgery-induced fibrosis may also exhibit increased ^68^Ga-FAPI uptake. Hence, differentiation between malignant and non-malignant diseases should not solely rely on ^68^Ga-FAPI uptake levels but should also consider other imaging findings and clinical evidence 58.

In terms of diagnosing lymph node metastases, ^68^Ga-FAPI generally shows improved sensitivity compared to ^18^F-FDG, as indicated by a higher SUVmax and TBR. However, some included studies found that the sensitivity of ^68^Ga-FAPI for metastatic lymph nodes was not significantly higher than that of ^18^F-FDG 33, 35, 37. Notably, Lin *et al.* concluded that when metastatic lymph nodes are very small (<5 mm), there could be a considerable number of missed diagnoses, leading to low sensitivity of ^18^F-FDG and ^68^Ga-FAPI for diagnosing lymph node infiltration 35. Yoshioka *et al.* suggested that ^18^F-FDG uptake in lymph node metastases originating from well-differentiated GC is higher than that in poorly differentiated tumours 54. Thus, in Çermik's study, regional metastatic lymph nodes could not be detected on ^18^F-FDG PET/CT in three cases (100%) of gastric SRCC but were clearly visualized on ^68^Ga-FAPI PET/CT 31. As is shown by **Figure 5,** ^18^F-FDG uptake was higher in patients with adenocarcinoma (including adenocarcinoma mixed with SRCC) than those with SRCC, but intense ^68^Ga-FAPI uptake was observed in both cancer types.

Moreover, inflamed lymph nodes may show high ^68^Ga-FAPI and ^18^F-FDG avidity, suggesting that ^68^Ga-FAPI may not be more specific than ^18^F-FDG for detecting lymph node metastasis 38. Interestingly, Chen *et al.* and Pang *et al.* reported that ^68^Ga-FAPI may be more suitable than ^18^F-FDG for differentiating reactive from metastatic lymph nodes, as reactive lymph nodes showed false-positive uptake of ^18^F-FDG and were correctly diagnosed by ^68^Ga-FAPI PET/CT (**Figure 8**) 39, 41. In addition, Miao *et al.* showed that dual-tracer (^68^Ga-FAPI + ^18^F-FDG) PET/CT did not significantly improve the diagnostic accuracy of regional lymph node metastasis compared with ^68^Ga-FAPI PET/CT alone, mainly due to the inability of ^18^F-FDG to detect additional metastatic lymph nodes or to reduce false-positive results for small and occult perigastric lymph nodes 36. Although our summarized information (**Table 3**) suggests that ^18^F-FDG may provide additional information to ^68^Ga-FAPI in diagnosing lymph node metastases, it's important to note that the sample size of these studies is small, and not all lesions were confirmed by histopathology. Overall, there are false-positive and false-negative results for ^18^F-FDG and false-positive results for ^68^Ga-FAPI in general regarding lymph node metastasis, so the true situation remains to be verified in studies with a large number of pathological findings 59.

Accurate staging of GC is crucial for determining appropriate treatment and prognosis. Overall, ^68^Ga-FAPI performs better than ^18^F-FDG in diagnosing distant metastases. Undoubtedly, ^68^Ga-FAPI PET imaging exhibits low background activity in the brain, heart, gastrointestinal tract, liver, and other tissues, making it superior for detecting tumour lesions 60. For instance, small metastases in the peritoneum, abdominal lymph nodes, liver and bone are challenging to detect on ^18^F-FDG PET, but the low background activity of ^68^Ga-FAPI PET imaging can visualize lesions with low ^18^F-FDG uptake, even those of very small sizes 29, 31, 37, 38, 41. However, in Miao's study, ^18^F-FDG PET/CT revealed three (42.9%) additional liver metastases without increased ^68^Ga-FAPI uptake, in addition to one (14.3%) false-positive liver lesion on ^68^Ga-FAPI PET/CT 36. Meanwhile, Miao *et al.* reported that in the diagnosis of distant metastases (*e.g.* bone, liver and lung metastases), the dual-tracer PET/CT (^68^Ga-FAPI+^18^F-FDG) was more sensitive than ^68^Ga-FAPI or ^18^F-FDG PET/CT alone. Researchers suggest that lesions that are negative on ^68^Ga-FAPI PET but positive on ^18^F-FDG PET may be due to the small size of the metastases 36. In these small lesions, the fibroblastic tissue proliferation reaction lags behind tumorigenesis and tumour activity changes. Therefore, there may be a delay in diagnosing lesions using ^68^Ga-FAPI PET/CT in the early stage of oncogenesis compared with ^18^F-FDG PET/CT 57.

Another concern is that both ^18^F-FDG and ^68^Ga-FAPI PET have physiological uptake in the ovaries and uterus of premenopausal women, indicating that both tracers have limitations 31, 36-38. In Chen's study, the specificity of ^68^Ga-FAPI was lower than ^18^F-FDG because ^68^Ga-FAPI PET/CT showed more false-positive visceral lesions, particularly in the liver and lungs (**Figure 8**) 39. Şahin *et al.* believed that excluding cirrhosis when enrolling patients may reduce false-positive uptake of ^68^Ga-FAPI PET due to inflammation and fibrosis of the liver parenchyma 29. In addition, false-positive ^68^Ga-FAPI uptake may also occur in conditions like myelofibrosis, arthritis, sarcoidosis, uterine fibroids, pneumonia, and esophagitis 39. Consequently, when using ^68^Ga-FAPI for staging, images should be interpreted cautiously to avoid misdiagnosis.

Patient-based analysis shows a comparable pooled sensitivity between ^68^Ga-FAPI and ^18^F-FDG for detecting bone lesions (0.93 *vs*. 1.00). Meanwhile, lesion-based analysis reveals a significantly higher sensitivity for ^68^Ga-FAPI (0.95 *vs*. 0.65). Despite ^68^Ga-FAPI detecting more bone metastases than ^18^F-FDG, it does not alter the staging or subsequent treatment regimens for stage IV cancer patients 37. Additionally, there is no significant difference in SUVmax and TBR derived from bone metastases between ^68^Ga-FAPI and ^18^F-FDG.

Research indicates that the extent of ^68^Ga-FAPI uptake and glycolytic activity of bone metastases might be related to the pathological type of the primary tumour 61. Our pooled results (**Figure 5**) suggest that metastatic bone lesions showed higher ^68^Ga-FAPI uptake than ^18^F-FDG in studies with high percentage of the SRCC subtype (*e.g.* 100% of SRCC in Chen's study and 45% of SRCC in Qin's study) 37, 39. In contrast, lower ^68^Ga-FAPI uptake in bone metastases was observed in studies with higher percentage of adenocarcinoma (*e.g.* 76% of adenocarcinoma in Gündoğan's study and 70% in Lin's study) 32, 35. Therefore, we speculate that the level of ^68^Ga-FAPI and ^18^F-FDG uptake in bone metastases may correlate with the subtype of the GC, *i.e*. tumours containing high component of CAFs may show greater uptake of ^68^Ga-FAPI and conversely, adenocarcinomas with a high density of active tumour cells may demonstrate higher ^18^F-FDG uptake.

Studies have shown that CAFs in the bone marrow can prompt dormant tumour cells to enter the neo-cellular cycle, thereby promoting the initiation and progression of bone metastases 62. Therefore, when the number of tumour cells or the activity of tumour cells are insufficient in the bone marrow, FAPI-PET may show great superiority over FDG-PET in the detection of bone metastases.

On the other hand, Wu *et al.* showed that both osteolytic and osteogenic lesions could be effectively detected using ^18^F-FDG and ^68^Ga-FAPI, but the detection rate of ^68^Ga-FAPI is significantly higher than that of ^18^F-FDG, especially for cranial metastases adjacent to the brain (due to the intense physiological FDG uptake in normal brain tissues) 61. However, in contrast to ^18^F-FDG, ^68^Ga-FAPI might demonstrate more false-positive bone lesions, thereby limiting its specificity. For instance, it may yield false positives in cases of degenerative osteophytes, arthritis, Schmorl nodes, fractures, and benign lesions associated with myelofibrosis 63-67. Since it is unlikely that most patients with multiple bone metastases will undergo biopsy confirmation, large prospective studies are required to fully ascertain the value of ^68^Ga-FAPI PET/CT in detecting bone metastases from GC.

Our pooled results revealed that the sensitivity of ^68^Ga-FAPI was notably superior to that of ^18^F-FDG in cases of peritoneal metastasis of GC. Additionally, the SUVmax and TBR of ^68^Ga-FAPI were significantly higher than those of ^18^F-FDG (**Figure 8**). This can be attributed to two main reasons. Firstly, ^18^F-FDG's accumulation in the intestine hinders the acquisition of clear images with high TBR in this region 68. In contrast, ^68^Ga-FAPI does not accumulate physiologically in the intestine, and its low background activity may assist in detecting peritoneal metastases 30, 36, 58, 67, 69. Secondly, severe fibrosis may occur after tumour invasion of the peritoneal tissue, providing a pathological basis for detecting lesions using ^68^Ga-FAPI PET imaging 25, 70, 71. Typically, peritoneal metastases are small, diffuse, and variable in appearance 72, 73. Both ^18^F-FDG PET/CT and MRI-DWI have shown a limited ability to detect sub-centimetre peritoneal implant foci 72. Cancerous foci larger than 1-2 mm in diameter require supportive stroma, and the stromal volume may exceed the tumour volume 74. Utilizing stroma-targeted ^68^Ga-FAPI may be more sensitive than glycolysis-targeted ^18^F-FDG in detecting small lesions with sufficient FAP expression 30, 36. Therefore, ^68^Ga-FAPI PET/CT could potentially be a promising tool for the non-invasive evaluation of peritoneal nodules and may guide the resection of these nodules. Nonetheless, as with ^18^F-FDG, inflammation may also result in false-positive uptake of ^68^Ga-FAPI 25.

The imaging modality used to evaluate primary and metastatic tumours of GC in some of the studies was PET/MR, mainly to take advantage of the added value of MRI, such as multiple sequences, in providing excellent soft tissue resolution and valuable functional information that can help interpret some lesions in the ovary, uterus, liver or bone 33, 37, 39, 40. In terms of radiotracer selection, most studies used ^68^Ga-FAPI-04, while Pang *et al.* used ^68^Ga-FAP-2286 and ^68^Ga-FAPI-46 25. They found lower physiological uptake of ^68^Ga-FAP-2286 than ^68^Ga-FAPI-46 in muscle, salivary glands, thyroid, and pancreas, but higher uptake of ^68^Ga-FAP-2286 than ^68^Ga-FAPI-46 in kidney, liver, and heart.

There are several limitations to our study that we have acknowledged. Firstly, the number of publications and patients included in this study was relatively limited, potentially impacting the reliability of our findings. Secondly, a majority of the studies we included were sourced from China, which might introduce inherent bias and differences in medical practices worldwide. Thirdly, the quality assessment results indicated some risk of bias in the studies we included, most of which were not randomized controlled trials, potentially affecting the overall quality of our research. Lastly, the most considerable source of heterogeneity within the included studies is the diagnostic method used to evaluate primary and recurrent tumours, lymph nodes, and distant metastases, which were mostly conventional imaging techniques such as contrast-enhanced CT/MRI or clinical follow-up information, rather than pathological evaluation.

In conclusion, ^68^Ga-FAPI demonstrated superior diagnostic accuracy in GC, overcoming the limitations of ^18^F-FDG. These limitations include poor detection of several pathological subtypes, a low detection rate of small tumours, and physiological uptake in the gastrointestinal tract, which obscures observation of lesions in the corresponding region. ^68^Ga-FAPI PET/CT showed a higher SUVmax and TBR than ^18^F-FDG in diagnosing primary tumours, lymph node infiltration, and distant metastatic lesions. This enhances physicians' diagnostic confidence and reduces missed diagnoses. Based on these advantages and features, ^68^Ga-FAPI may potentially replace ^18^F-FDG in future GC applications.

## Supplementary Material

Supplementary figures and table.

## Figures and Tables

**Table 1:** Basic characteristic information for the included studies

Year	Author	Ref	Patients' Origin	Study design	Major research objectives	N of pts	Types of patients	*Age (years)	Male / Female	Radiotracer	imaging modalities (FAPI)	Reference standard	Extracted data of GC	SRCC (%)	*Interval time (days)	Analysis Method	duration time (min)		Quantitative Assessment Metrics
2021	Pang Y	25	China	Re	Comparing the diagnostic value of FAP-04 and FDG for initial staging and restaging of gastrointestinal malignancies	35	4 types of gastrointestinal malignancies (18 GC + 2 GSRCC)	64 (53-68)	18/17	FAPI-04 *vs*. FDG	PET/CT	pathology	20 GC (11 initial staging + 9 restaging)	2/20 (10.0%)	2 (1-6)	V + Sq	60 / 60		SUVmax
2021	Şahin E	29	Turkey	Re	Comparing the diagnostic value of FAP-04 and FDG for liver metastases of gastrointestinal malignancies	31	4 types of gastrointestinal malignancies (4 GC)	61.9 ± 10.9	19/12	FAPI-04 *vs.* FDG	PET/CT	pathology + follow up	4 GC for detecting liver metastases	NR	≥ 14	V + Sq	45 / 60		SUVmax *&* TBR
2021	Zhao L	30	China	Re	Comparing the diagnostic value of FAP-04 and FDG for metastatic peritoneal carcinoma	46	10 types of cancer (9 gastric Ade + 4 GSRCC)	57 (32-80)	14/32	FAPI-04 *vs.* FDG	PET/CT	pathology + follow up	13 GC for detecting peritoneal metastases	4/13 (30.8%)	≤ 7	V + Sq	60 / 60		SUVmax
2022	Çermik TF	31	Turkey	Pro	Comparing the diagnostic value of FAP-04 and FDG for initial staging and restaging of various types of cancer	42	22 types of cancer (3 gastric Ade + 3 GSRCC)	58.5 (31-84)	26/16	FAPI-04 *vs.* FDG	PET/CT	pathology + follow up	6 GC for initial staging and restaging	3/6 (50.0%)	≤ 7	V + Sq	60 / 60		SUVmax
2022	Gündoğan C	32	Turkey	Pro	Comparing the diagnostic value of FAP-04 and FDG for initial staging of GC	21	21 gastric Ade (5 GSRCC)	61 (40-81)	12/9	FAPI-04 *vs.* FDG	PET/CT	pathology	21 GC (15 initial staging+6 restaging)	5/21 (23.8%)	≤ 7	V + Sq	60 / 60		SUVmax *&* TBR
2022	Jiang D	33	China	Re	Comparing the diagnostic value of FAP-04 and FDG for initial staging of GC in 2 centers	38	38 GC (7 GSRCC)	67.5 (25-86)	29/9	FAPI-04 *vs.* FDG	PET/CT *&* PET/MR	pathology	38 GC for initial staging	7/38 (18.4%)	1.6 ± 0.8	V + Sq	60 / 60		SUVmax *&* TBR
2022	Kuten J	34	Israel	Pro	Comparing the diagnostic value of FAP-04 and FDG for initial staging of GC	13	13 gastric Ade (4 GSRCC)	70 (35-87)	6/7	FAPI-04 *vs.* FDG	PET/CT	pathology + follow up	13 GC (10 initial staging + 3 restaging)	4/13 (30.8%)	6 (1-23)	V + Sq	60 / 60		SUVmax *&* TBR
2022	Lin R	35	China	Pro	Comparing the diagnostic value of FAP-04 and FDG for initial staging of GC	56	56 GC (17 GSRCC)	63.8 ± 14.9	40/16	FAPI-04 *vs.* FDG	PET/CT	pathology + follow up	56 GC (45 initial staging + 11 restaging)	17/56 (30.4%)	≤ 7	V+ Sq	5-71 /5-71		SUVmax *&* TBR
2022	Miao Y	36	China	Pro	Comparing the diagnostic value of FAP-04 and FDG for initial staging of GC	62	62 GC (27 PCC, 35 non-PCC)	64 (24-75)	44/18	FAPI-04 *vs.* FDG	PET/CT	pathology + follow up	62 GC for initial staging	NR	≤ 9	V + Sq	30-60 / 60-90	SUVmax	
2022	Qin C	37	China	Pro	Comparing the diagnostic value of FAP-04 and FDG for initial staging of GC	20	20 GC (4 GSRCC, 5 with partial GSRCC)	56.0 (29-70)	9/11	FAPI-04 *vs.* FDG	PET/MR	pathology + follow up	20 GC (14 initial staging + 6 restaging)	9/20 (45.0%)	≤ 7	V + Sq	30-60 / 60	SUVmax *&* TBR	
2022	Zhang S	38	China	Re	Comparing the diagnostic value of FAP-04 and FDG for initial staging of GC	25	25 gastric Ade (5 with partial GSRCC)	56 ± 12	12/13	FAPI-04 *vs.* FDG	PET/CT	pathology + follow up	25 GC (17 initial staging + 8 restaging)	5/25 (20%)	≤ 7	V + Sq	60 / 60	SUVmax	
2023	Chen H	39	China	Re	Comparing the diagnostic value of FAP-04 and FDG for initial staging and restaging of GSRCC in a multicenter	34	34 GSRCC	51 (25-85)	16/18	FAPI-04 *vs.* FDG	PET/CT *&* PET/MR	pathology + follow up	34 GC (22 initial staging + 12 restaging)	34/34 (100%)	2 (1-7)	V + Sq	60 / 60	SUVmax *&*TBR	
2023	Du T	40	China	Pro	Comparing the diagnostic value of FAP-04 and FDG for the preoperative diagnosis of GC	40	40 gastric tumours (23 gastric Ade + 13 GSRCC + 4 benign)	40	32/18	FAPI-04 *vs.* FDG	PET/MR	pathology	40 gastric tumours for initial diagnosis and preoperative staging	13/40 (32.5%)	> 2	V + Sq	40/40	SUVmax *&* SULmax	
2023	Pang Y	41	China	Pro	Comparing the diagnostic value of FAP-2286 with FAPI-46 and FDG for initial staging and restaging of various types of cancer	64	15 types of cancer (6 GC)	57.5 (32-85)	38/26	FAP-2286 *vs.* FDG *&* FAPI-46	PET/CT	pathology + follow up	6 GC for initial staging	NR	≤ 7	V + Sq	60 / 60	SUVmax *&* TBR	
																			

Ref: reference; N of pts: number of patients; Re: retrospective; Pro: prospective; NR: not report; Duration time: duration time after injection (FAPI / FDG); V: visual; Sq: semi-quantitative. FAPI: ^68^Ga-FAPI; FDG: ^18^F-FDG; GC: gastric cancer; GSRCC: gastric signet ring cell carcinoma; Ade: adenocarcinoma; PCC: poorly cohesive carcinoma; SUVmax: maximum standardized uptake value; SULmax: maximum fat removal standard uptake value. *Age (years) and *Interval time (days) counted as Median *&* range/ Mean *&* SD.

**Table 2:** Summary of the pooled sensitivity of ^68^Ga-FAPI and ^18^F-FDG PET/CT for GC

		^68^Ga-FAPI		^18^F-FDG	
Analysis type	Lesion site	Pooled sensitivity	95% CI	Pooled sensitivity	95% CI
Patient-based	Primary tumour	0.95	0.91─0.97	0.72	0.67─0.78
	Recurrent tumour	1.00	0.89─1.00	0.48	0.29─0.68
	Lymph node metastases	0.72	0.59─0.82	0.47	0.32─0.62
	Distant metastases	0.83	0.59─0.96	0.50	0.26─0.74
	^*^Bone metastases	0.93	0.66─1.00	1.00	0.77─1.00
	Peritoneal metastases	0.98	0.92─1.00	0.47	0.34─0.59
Lesion-based	Lymph node metastases	0.64	0.58─0.70	0.32	0.27─0.38
	Distant metastases	0.96	0.94─0.97	0.45	0.41─0.48
	Bone metastases	0.95	0.91─0.98	0.65	0.57─0.71
	Peritoneal metastases	1.00	0.98─1.00	0.31	0.25─0.37

^*^Including the studies: 2022 Gündoğan C ^68^Ga-FAPI [sensitivity: 4_patient_/4_patient_ (117 lesions)] *vs.* ^18^F-FDG [sensitivity: 4_patient_/4_patient_ (101 lesions)]; 2022 Qin C ^68^Ga-FAPI [sensitivity: 3_patient_/3_patient_ (12 lesions)] *vs.* ^18^F-FDG [sensitivity: 3_patient_/3_patient_ (4 lesions)]

**Table 3:** Summary of diagnostic accuracy of ^68^Ga-FAPI, ^18^F-FDG PET/CT, and dual-tracer PET/CT in detecting metastatic lesions of GC

	^68^Ga-FAPI			^18^F-FDG			Dual-tracer	
	Sensitivity	Specificity		Sensitivity	Specificity		Sensitivity	Specificity
Recurrence detection								
2022 Gündoğan C	1.00 (4/4)	0.50 (1/2)		1.00 (4/4)	1.00 (2/2)		1.00 (4/4)	1.00 (2/2)
Lymph node metastases								
2022 Miao Y	0.64 (7/11)	0.89 (8/9)		0.55 (6/11)	0.78 (7/9)		0.73 (8/11)	0.78 (7/9)
2022 Qin C	0.88 (14/16)	1.00 (4/4)		0.75 (12/16)	1.00 (4/4)		0.94 (15/16)	1.00 (4/4)
Distant metastases								
2022 Miao Y	0.76 (26/34)	NR		0.74 (25/34)	NR		0.97 (33/34)	NR
Bone metastases								
2022 Miao Y	0.67 (2/3)	NR		1.00 (3/3)	NR		1.00 (3/3)	NR
Liver metastases								
2022 Miao Y	0.57 (4/7)	NR		0.86 (6/7)	NR		1.00 (7/7)	NR
Lung metastases								
2022 Miao Y	0.00 (0/2)	NR		1.00 (2/2)	NR		1.00 (2/2)	NR

NR: not report.

## Abbreviations

FAPI: fibroblast-activation protein inhibitor
FDG: fluorodeoxyglucose
PET/CT: positron emission tomography/computed tomography
PET/MR: positron emission tomography/magnetic resonance
SUVmax: maximum standardized uptake value
TBR: tumor-to-background ratios
MD: mean difference
GC: gastric cancer
SRCC: signet ring cell carcinoma
CAFs: cancer-associated fibroblasts
AUC: area under the curve
SROC: summary receiver-operation characteristic
