# Supplementary material for: Evaluation of FAPI PET imaging in gastric cancer: a systematic review and meta-analysis
Source: Theranostics. 2023 Aug 21;13(13):4694–710. doi: 10.7150/thno.88335 (PMC10465231; doi:10.7150/thno.88335)
Supplement: Supplementary file 1 — Supplementary figures and table. [file thnov13p4694s1.pdf]

Table S1 Detailed patient-based and lesion-based diagnostic data for each study

		^{68}Ga-FAPI					^{18}F-FDG			
		TP	FP	FN	TN		TP	FP	FN	TN
**Patient-based analysis**										
Primary tumor	2023 Pang Y	6	NA	0	NA	2023 Pang Y	3	NA	3	NA
	2023 Chen H	16	NA	6	NA	2023 Chen H	4	NA	18	NA
	2022 Miao Y	56	NA	6	NA	2022 Miao Y	48	NA	14	NA
	2022 Zhang S	18	0	1	6	2022 Zhang S	13	1	6	5
	2022 Lin R	45	NA	0	NA	2022 Lin R	44	NA	1	NA
	2022 Gündoğan C	15	NA	0	NA	2022 Gündoğan C	13	NA	2	NA
	2022 Kuten J	10	NA	0	NA	2022 Kuten J	5	NA	5	NA
	2022 Jiang D	38	NA	0	NA	2022 Jiang D	31	NA	7	NA
	2022 Qin C	14	NA	0	NA	2022 Qin C	10	NA	4	NA
	2021 Pang Y	11	NA	0	NA	2021 Pang Y	4	NA	7	NA
	2023 Du T	34	3	2	1	2023 Du T	26	3	10	1
Reccurence tumor	2023 Chen H	7	NA	0	NA	2023 Chen H	2	NA	5	NA
	2022 Lin R	11	NA	0	NA	2022 Lin R	9	NA	2	NA
	2022 Gündoğan C	4	1	0	1	2022 Gündoğan C	NA	NA	NA	NA
	2021 Pang Y	9	NA	0	NA	2021 Pang Y	2	NA	7	NA
Lymph node metastases	2022 Qin C[a]	11	NA	0	NA	2022 Qin C	10	NA	1	NA
	2022 Miao Y	7	1	4	8	2022 Miao Y	6	2	5	7
	2022 Lin R	5	NA	6	NA	2022 Lin R	5	NA	6	NA
	2022 Gündoğan C	20	1	0	0	2022 Gündoğan C	NA	NA	NA	NA
	2022 Kuten J	3	NA	0	NA	2022 Kuten J	2	NA	1	NA
	2022 Jiang D	6	1	4	13	2022 Jiang D	5	1	5	13
	2023 Du T	7	5	5	12	2023 Du T	4	3	8	14
Distant metastases	2022 Zhang S	12	NA	2	NA	2022 Zhang S	7	NA	7	NA
	2021 Şahin E	3	NA	1	NA	2021 Şahin E	2	NA	2	NA
Bone metastases	2022 Miao Y	2	NA	1	NA	2022 Miao Y	3	NA	0	NA
	2022 Lin R	4	NA	0	NA	2022 Lin R	4	NA	0	NA
	2022 Gündoğan C	4	NA	0	NA	2022 Gündoğan C	4	NA	0	NA
	2022 Qin C	3	NA	0	NA	2022 Qin C	3	NA	0	NA
peritoneal metastases	2022 Miao Y	11	NA	1	NA	2022 Miao Y	5	NA	7	NA
	2022 Lin R	13	NA	0	NA	2022 Lin R	9	NA	4	NA
	2022 Gündoğan C	10	NA	0	NA	2022 Gündoğan C	4	NA	6	NA
	2022 Kuten J	5	NA	0	NA	2022 Kuten J	0	NA	5	NA
	2022 Qin C	10	NA	0	NA	2022 Qin C	4	NA	6	NA
	2021 Zhao L	13	NA	0	NA	2021 Zhao L	7	NA	6	NA
**Lesion-based analysis**										
Primary tumor	2022 Lin R	45	0	1	0	2022 Lin R	44	0	2	0
Lymph node metastases	2023 Chen H	59	11	18	405	2023 Chen H	18	10	59	406
	2022 Zhang S	75	2	0	8	2022 Zhang S	32	4	43	6
	2022 Lin R	20	0	84	521	2022 Lin R	16	12	88	509
	2022 Kuten J	17	NA	0	NA	2022 Kuten J	16	NA	1	NA
Distant metastases	2023 Chen H	207	13	15	11	2023 Chen H	86	7	136	17
	2022 Miao Y	26	2	8	292	2022 Miao Y	25	2	9	292
	2022 Zhang S	275	NA	8	NA	2022 Zhang S	122	NA	161	NA
	2022 Lin R	237	NA	0	NA	2022 Lin R	114	NA	123	NA
Bone metastases	2023 Chen H	113	NA	9	NA	2023 Chen H	65	NA	57	NA
	2022 Lin R	64	NA	0	NA	2022 Lin R	55	NA	9	NA
Peritoneal metastases	2023 Chen H	59	NA	0	NA	2023 Chen H	20	NA	39	NA
	2022 Lin R	159	NA	0	NA	2022 Lin R	47	NA	112	NA

[a]Diagnostic data only included the abdominal lymph nodes. NA: Not available.

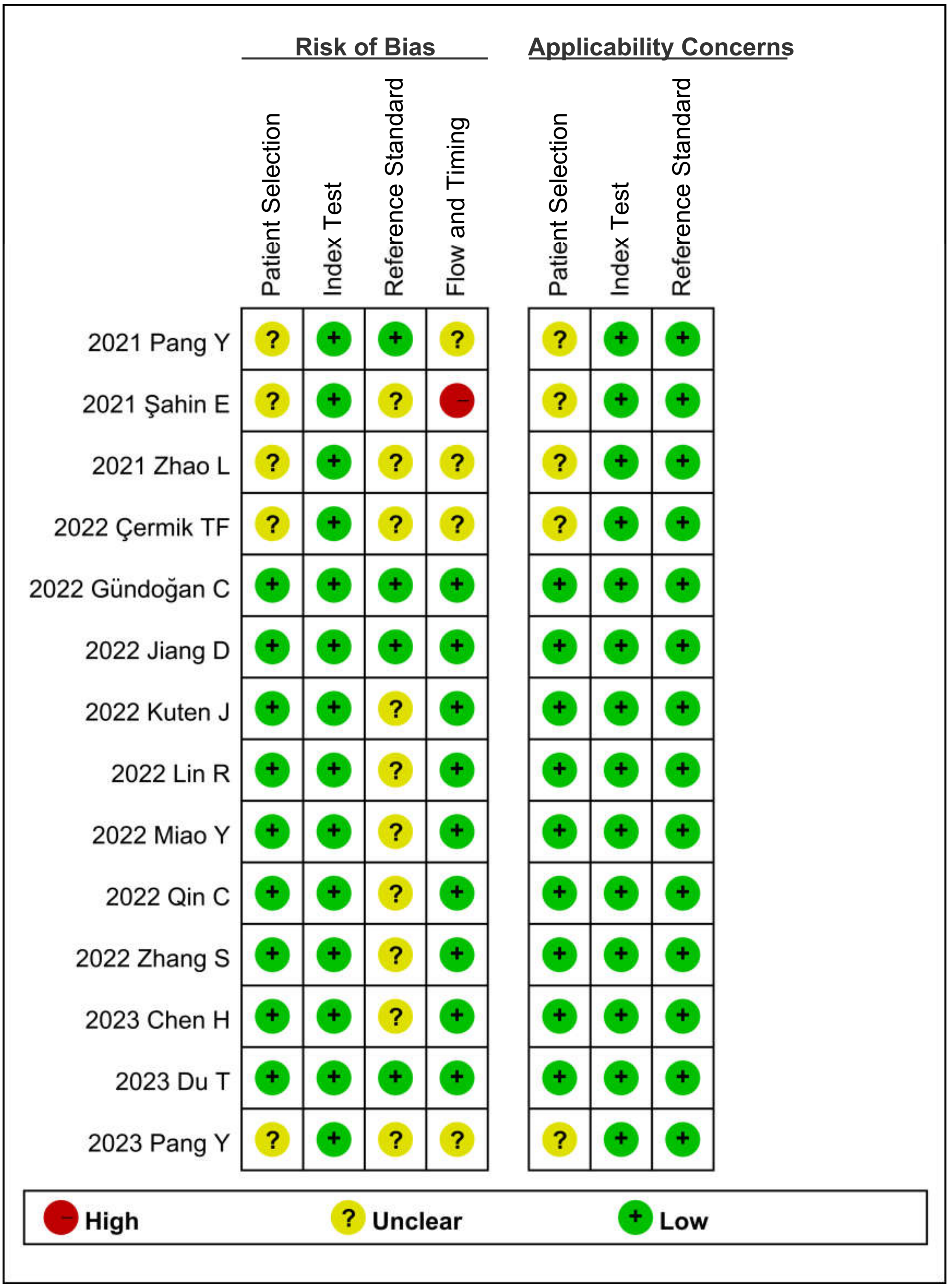

Figure S1 Quality assessment of each study.

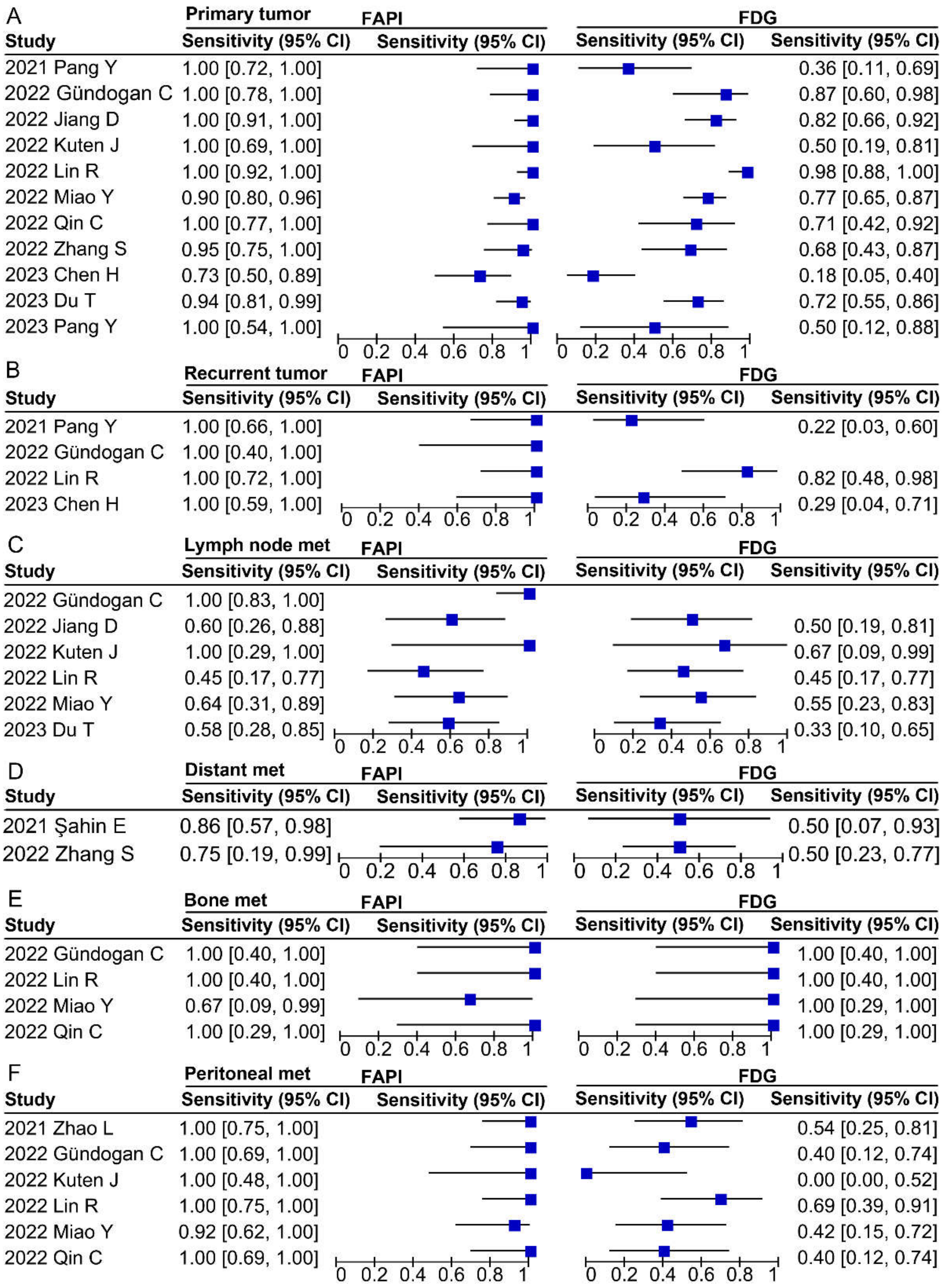

Figure S2 Comparison of diagnostic sensitivity between ^{68}Ga-FAPI and ^{18}F-FDG PET/CT for primary tumours (A), recurrent tumours (B), lymph node metastases (C), distant metastases (D), bone metastases (E), and peritoneal metastases (F) for each included study (patient-based analysis)

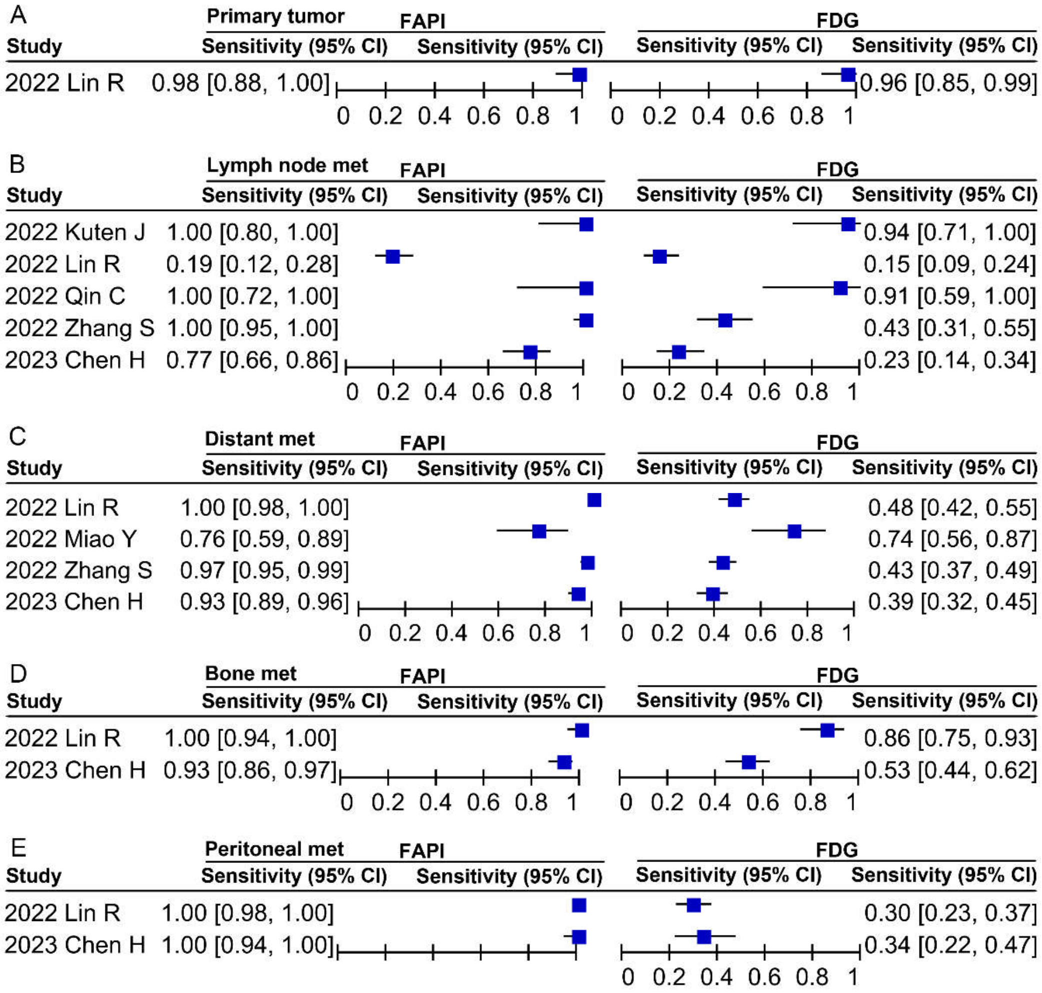

Figure S3 Comparison of diagnostic sensitivity between ^{68}Ga-FAPI and ^{18}F-FDG PET/CT for primary tumour (A), lymph node metastases (B), distant metastases (C), bone metastases (D), and peritoneal metastases (E) for each included study (lesion-based analysis)
